# Synergistic Cytotoxic Effects of *Ganoderma lucidum* and Bacillus Calmette Guérin on Premalignant Urothelial HUC-PC Cells and Its Regulation on Proinflammatory Cytokine Secretion

**DOI:** 10.1155/2012/147896

**Published:** 2012-08-28

**Authors:** John Wai-man Yuen, Mayur-Danny I. Gohel, Chi-fai Ng

**Affiliations:** ^1^School of Nursing, The Hong Kong Polytechnic University, Hung Hom, Hong Kong; ^2^Department of Medical Science, Tung Wah College, Homantin, Hong Kong; ^3^Department of Surgery, The Chinese University of Hong Kong, Shatin, Hong Kong

## Abstract

Bacillus Calmette-Guérin (BCG) is conventionally used as an adjuvant immunotherapy to reduce the recurrence of bladder cancer. To address the issues of efficacy and safety, an ethanol extract of *Ganoderma lucidum* (*GLe*) was evaluated for its interaction with BCG. In a model of premalignant human uroepithelial cells (HUC-PC), *GLe* exerted immediate cytotoxic effects while BCG showed a delayed response, given that both were immunological active in inducing the secretion of interleukin (IL)-6, IL-8, and monocyte chemotactic protein-1 (MCP-1). Synergistic cytotoxic effects were observed when cells were either coincubated with both drugs or firstly preincubated with *GLe*. Synergism between *GLe* and BCG was demonstrated to achieve a complete cytostasis in 24 hours, and such effects were progressed in the subsequent 5 days. However, the pretreatment of *GLe* resulted in suppression of IL-6, IL-8, and MCP-1 secretions without affecting the cytotoxicity. Given that numerous proinflammatory cytokines are associated with the high side effects toll of BCG, results herein suggested the potential implications of GL to supplement the BCG immunotherapy in bladder cancer, for better efficacy and reducing side effects.

## 1. Introduction

Transitional cell carcinoma (TCC) of the urinary bladder is known for its high recurrence rate up to 80% if patients are treated by surgical ablation alone [1]. Despite the mechanism of action is not fully understood, Bacillus Calmette-Guérin(BCG) has been used for over 30 years as a prophylactic agent for preventing the TCC recurrence. Intravesical instillation of BCG following transurethral resection (TUR) has shown an overall effectiveness in diminishing 30–40% of the recurrence and progression [2]. Such effects were relied on the mycobacterium nature of BCG, which was able to trigger local nonspecific immune response through cytokines secretion and recruitment of immune cells to the bladder wall [3]. Several cytokines, some are sourced from urothelial cells, were detected in patients' voided urine upon BCG instillation. In culture experiments, BCG was shown to be active in stimulating tumor-necrosis-factor- (TNF-) related apoptosis in accordance with the production of cytokines including interleukin (IL)-1, IL-6, IL-8, and TNF-*α*, whereas the activity was tumor grade dependent [4]. Besides, BCG also leads to a high side effect toll of 90% from local cystitis and haematuria, allergic reactions to severe systemic infection [5]. Controversially, the immunological activities induced by BCG, especially some of the cytokines, were suggested as the side effects cause [6, 7]. Therefore, a new chemopreventive regimen with better efficacy and lesser side effects is demanded.

The recurrence of TCC was associated with the presence of papillary multifocality. This is explained by the “field cancerization hypothesis” and the “clonal seeding theory” that residual adverse cells at premalignant stage could not be completely removed by surgery, and thus they are readily being transformed by any stimulation [1]. In this relation, for evaluating potential bladder prophylactic agents, we postulated the importance of utilizing a human uroepithelial (HUC-PC) cell line, which carries the premalignant features of being sensitive to bladder carcinogens for undergoing tumorigenic transformation [8]. *Ganoderma lucidum*, an ancient medicinal mushroom belonging to the family of Ganodermataceae of Aphyllophorales, has been widely used for promotion of health and longevity. Its popularity and perceived health benefits have also prompted the usage by cancer patients. With polysaccharides and triterpenes as principle bioactive constituents, a range of scientific evidence from *in vitro* to animals and humans *in vivo* has been discovered for supporting the antitumorigenicity of *G. lucidum* in cancer of different origins [9]. In the past years, some works have been done to explore the chemopreventive properties of this mushroom on TCC. Antiproliferation was demonstrated on TCC cells with different degrees of malignancy, from premalignant to high-grade invasive [10]. In the premalignant HUC-PC cells, growth inhibition via G2/M phase cell cycle arrest and apoptosis was shown by ethanol extract of* G. lucidum *(*GLe*), in addition to the suppression of carcinogen 4-aminobiphenyl-mediated cell migration and telomerase activity [10, 11]. More recently, *GLe* was found to be immunologically active to induce secretion of an array of cytokines secretion and neutrophilic migration, in the culture of HUC-PC cells [12]. Such properties generate the next question to ask whether *GLe* could be synergistic with BCG in elimination of adverse cells of TCC. By using the HUC-PC cell model, a pilot study was conducted and indicated that BCG was noncytotoxic in 24 hours after incubation but stimulated a dose-dependent IL-6 production via nuclear factor-kappa B (NF-*κ*B) pathways. In the present study, interaction between *GLe* and BCG was evaluated using two treatment approaches: combination therapy and a pretreatment of *GLe* followed by BCG treatment. Cell viability and expression of cytokines (T-helper (Th)1, Th2 cytokines and chemokines) in response to the treatments were investigated as outcome measures. Furthermore, the cytotoxicities induced by test agents were assessed in a prolonged period, in order to confirm the persistence or delay of response. 

## 2. Materials and Methods

### 2.1. *G. lucidum* Extract and Chemicals

A proprietary extract consisting of *G. lucidum* fruiting bodies and cracked spores, branded ReishiMax GLP, was purchased from Pharmanex Inc. (Hong Kong). The active ingredients of the product were standardized to 13.5% polysaccharides (*β*-1,3-glucans) and 6% triterpene (ganoderic acids and others), which is the highest extractable levels, whereas the remaining 80% consisted of nucleosides, fatty acids, and amino acid according to the manufacturer's technical bulletin. The powdered *G. lucidum* from capsules was re-extracted as previously described [11]. Briefly, capsule contents were dissolved in 95% HPLC grade ethanol for 30 minutes, and the supernatant was further extracted by successive sonication using absolute ethanol. The reextracts were filtered (through 0.45 *μ*m polypropylene filter) and dried under reduced pressure to yield a water-insoluble extract (*GLe*) as brown-colored powder. For experiments, the *GLe* was dissolved in absolute ethanol (with final solvent concentration below 0.01% vol/vol), and then immediately diluted to 200 *μ*g/mL *GLe* assay media stock with complete media before adding into cultures. Immucyst BCG was sourced from Aventis (Toronto, Canada). The whole vial of attenuated BCG (81 mg dry weight containing 5% monosodium glutamine) was reconstituted with 3 mL of the accompanying diluents to make a stock solution containing a minimal 6.6 × 10^8^ colony forming units (CFU). The stock solution was further diluted with complete medium into the working concentrations for culture. The Limulus Amebocyte Lysate (LAL) endpoint chromogenic kit assay (CAPE CO, E. Falmouth, MA, USA) was performed to detect lipopolysaccharides (LPS) contamination in the *GLe* and BCG. In this assay, Glucashield buffer (CAPE COD) was used to reconstitute pyrochrome to inhibit possible (1, 3)-*β*-D-glucan present in samples, and thus avoiding potential interference. Aseptic techniques were strictly applied throughout the procedures.

### 2.2. Cell Culture and Treatment

**Figure 1:**
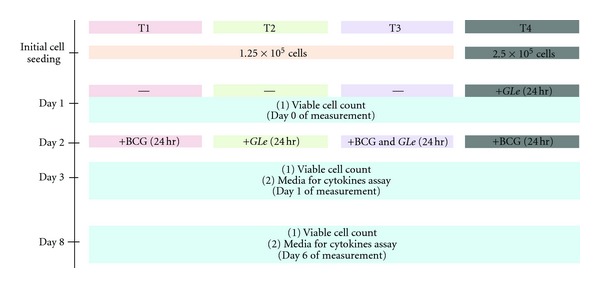
The treatment schedules for experiments, illustrating 4 treatments (T1–T4) were tested. A 24-hour incubation was allowed for stabilizing the culture environment after seeding the cells. The initial cell seeding numbers of T1–T3 were halves of T4, because cell cultures for T1–T3 were maintained for an additional 24 hours in complete media, in order to align initial cell numbers for all treatment groups before the first day sampling (Day 0 as baseline), that is, approximately 2.5 × 10^5^ cells since doubling time for the HUC-PC cells was 24 hours. For T4, a range of *GLe* concentrations was administrated before the baseline, and this complete medium in the absence of *GLe* (0 *μ*g/mL) was used as the baseline for this treatment group. Subsequent samples were collected on Day 1 (Day 3 after initial cell seeding) and Day 6 (Day 8 after initial cell seeding) following different treatments.

The HUC-PC cell line (provided by Dr. Rao from the University of California, Los Angeles, USA) was cultured in F12-Ham enhanced Dulbecco's Modified EaGLe's Medium (Sigma, St. Louis, USA) with 1% penicillin (10,000 *μ*g/mL) and streptomycin (10 mg/mL) and 10% fetal bovine serum (Gibco Brl Island, New York, USA). All cultures were maintained at 37°C in a water-saturated atmosphere containing 5% CO_2_. The effects of BCG were tested with or without *GLe* by using 4 different treatment schedules (2 of them were for synergism) illustrated in Figure 1. Logarithmically growing cells were plated in 6-well culture plates at 1.25 × 10^5^ cells per well for treatment 1–3 and 2.5 × 10^5^ cells per well for treatment 4. The different cell seeding density in treatment 4 was set to obtain the closest baseline cell numbers as other treatment schedules when corresponding treatment initiated. Treatment 1 and 2 were used to test BCG (at 0, 1.2 × 10^7^, 2.4 × 10^7^, and 4.8 × 10^7^ CFU) and *GLe* (0, 40, 80, 100 *μ*g/mL), respectively. Treatment 3 was the combination test for BCG (at fixed concentration of 1.2 × 10^7^ CFU) with different concentrations of *GLe* listed above. Whilst treatment 4 was the *GLe*-pretreatment protocol that a 24-hour BCG treatment was given following the 24-hour *GLe *pretreatment. For all treatment schedules, culture media and cells were harvested when the corresponding treatment was completed, and used for measuring the cytokine levels and viable cell count (day 1), respectively. In parallel experiments, cultures were maintained after removal of assay media and pure complete media (in the absence BCG or *GLe*) was refreshed every 2 days to ensure nutrient supply. On day 6 (5 days after retraction of treatment), cells were harvested for viable cell count. Cytokine levels were not determined on day 6 because preliminary data indicated that vast cytotoxicities have appeared in many of the treated cultures that number of viable cells was insufficient for cytokines production. Cells treated with solvent media, that is, 0.1% v/v ethanol for *GLe* and 33% v/v diluents for BCG were used as control experiments. 

### 2.3. Cytotoxicity Assay

Cell viability was assessed by the automated Beckman Coulter Vi-CELL XR cell viability analyzer with its reagent pack (Miami, FL, USA). All cell counting results were verified between the manual and automated methods and expressed as the viable cell numbers for interpretation.

### 2.4. FlowCytomix for Cytokines Measurement

The FlowCytomix (Bender MedSystems, Austria) human Th1/Th2 11plex and human chemokine 6plex kits were used for cytokines measurement. In total, 15 cytokines: interferon-(IFN)-*γ*, IL-1*β*, IL-2, IL-4, IL-5, IL-6, IL-8, IL-10, IL-12p70, tumor necrosis factor (TNF)-, TNF-*β*, monocyte chemotactic protein-1 (MCP-1), granulocyte colony stimulating factor (G-CSF), monokine induced by interferon gamma (MIG), macrophage inflammatory protein(MIP)-1*α*, and MIP-1*β* were measured, whereas IL-8 was overlapped in both kits. The Cytomics FC500 Flow cytometer (Beckman Coulter, Miami) equipped with CXP software version 2.2 was used. The forward scatter measurements were collected at 1–8 degrees. Standard mixture was prepared for each kit by mixing the standard of each analyte. The setup beads and standard mixture with highest phycoerythrin (PE) signal (FL-2 585/42 BP for detection) were used to set up the flow cytometry. Dilutions of each standard mixture (total 7 concentrations) were used to create the standard curve. And concentrations of each cytokine in the harvest media were analyzed and read against the corresponding standard curve using the FlowCytomix Pro software (Version 2.2, Bender Medsystem, Austria). Coefficients of variation <15% and absence of mavericks were achieved for reliable standard curves.

### 2.5. Statistical Analysis

All assays were performed in triplicate for reproducibility. Descriptive statistics with mean ± standard deviation were used to summarize the results. Differences between means were determined using the one-way analysis of variance (ANOVA) followed by Dunnett's test (GraphPad Prism version 3.0 for Windows, San Diego, Ca, USA), whereas statistical significance was sought at two-tailed *P*-value of 0.05. 

## 3. Results

### 3.1. Differentiated Properties of BCG and *GLe* on HUC-PC Cells

**Figure 2:**
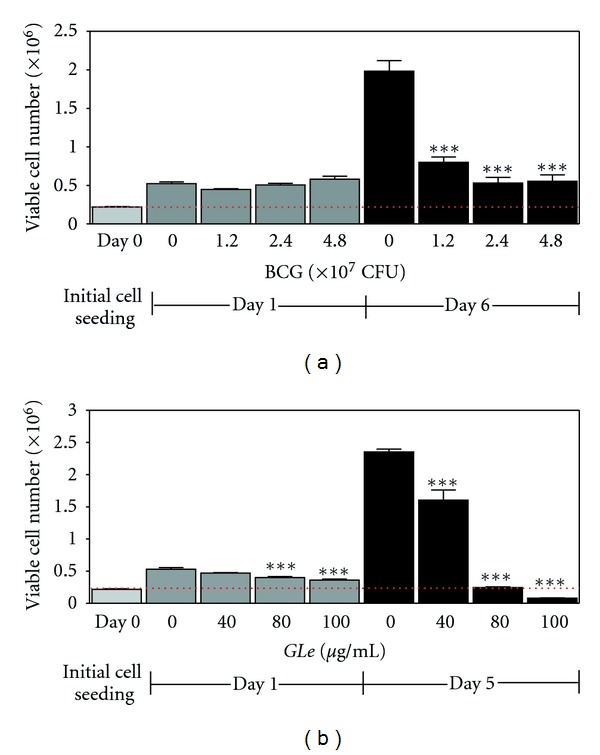
Showing the immediate and progressive cytotoxic effects exhibited by (a) BCG on day 1 (*F* = 4.908; statistically nonsignificant) and day 6 (*F* = 51.30; ***P < 0.001), and (b) *GLe* on day 1 (*F* = 18.69; ***P < 0.001) and day 6 (*F* = 118.5; ***P < 0.001). Results of BCG and *GLe* were statistically compared with the solvent media control according to the corresponding schedule (i.e., day 1 versus Day 1 and Day 6 versus Day 6), at 0 CFU containing BCG diluents (33% v/v) and at 0 *μ*g/mL containing ethanol (0.1%; v/v), respectively. All experiments were performed in triplicate for reproducibility. *F* value of each treatment group was determined by one-way ANOVA, and Dunnett's posttest was followed to determine *P* values.

Immediately after the 24-hour incubation, no cytotoxicity was demonstrated by BCG, except at 1.2 × 10^7^ CFU, about 15% of viable cell number was reduced (statistically nonsignificant). Nonetheless, a dose-dependent growth inhibition was observed 5 days after the BCG retraction (Figure 2(a)). In contrast, remarkable dose-dependent growth suppression was observed immediately after the 24-hour incubation with *GLe*, whereas the cytostatic effects were further progressed to become more significant in the next 5 days after *GLe* withdrawal (Figure 2(b)). Consistent with previous findings [11, 13], *GLe* at 80 *μ*g/mL was shown to be growth suppressing that was able to maintain the cell culture at initial seeding cell density for at least 5 days following *GLe* treatment (as shown in Figure 2(b)) but became cytotoxic to eliminate the adverse cells when concentration reached 100 *μ*g/mL or above. 

Cytokine secretion following different treatment schedules were summarized in Table 1. Amongst the 15 cytokines tested, only IL-6, IL-8, and MCP-1 were detectable in the cultures of HUC-PC cells without treatment. BCG (treatment 1) and *GLe* (treatment 2) exhibited similar activities in inducing IL-6 and IL-8 production (*P *< 0.001), reducing MCP-1 levels (*P* < 0.001 for all *GLe *concentrations but *P* < 0.001 for BCG at 1.2 × 10^7^ CFU only). The increase or decrease of cytokines demonstrated by *GLe* was in clear dose-dependent manner, whereas *GLe* has also stimulated a trace amount of IL-2 production but statistically nonsignificant. On the contrary, the BCG-mediated cytokines were not dose dependent. 

### 3.2. Synergistic Cytotoxicity between *GLe* and BCG

**Figure 3:**
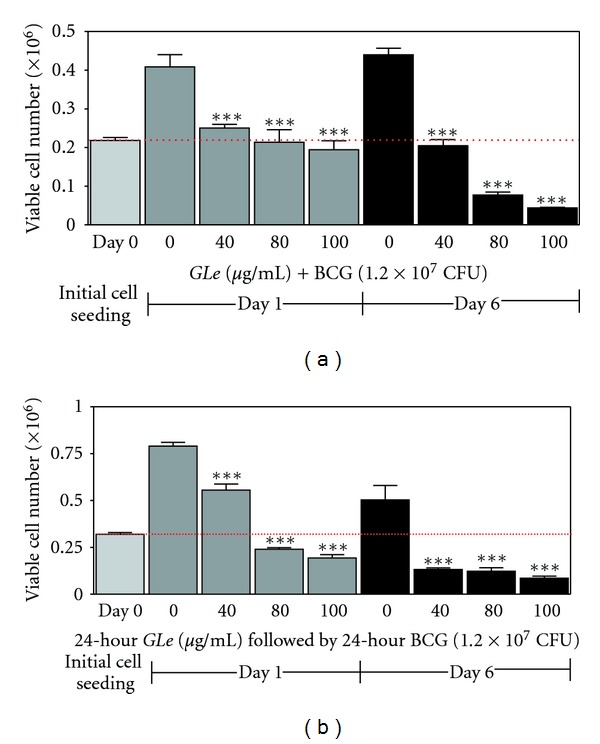
Showing the immediate and progressive cytotoxic effects exhibited by (a) coincubation of *GLe* with BCG on day 1 (*F* = 14.14; ***P < 0.01) and day 6 (*F* = 217.6; ***P < 0.001), and (b) pretreatment of *GLe* followed by BCG on day 1 (*F* = 169.8; ***P < 0.001) and day 6 (*F *= 30.52; ***P < 0.01). For all test conditions, BCG concentration was fixed at 1.2 × 10^7^ CFU for testing different concentrations of *GLe*. Results were statistically compared with control at 0 *GLe* concentration but treated with BCG at 1.2 × 10^7^ CFU, according to the corresponding schedule (i.e., day 1 versus day 1 and day 6 versus day 6). All experiments were performed in triplicate for reproducibility. *F* value of each treatment group was determined by one-way ANOVA, and Dunnett's posttest was followed to determine *P* values.

**Figure 4:**
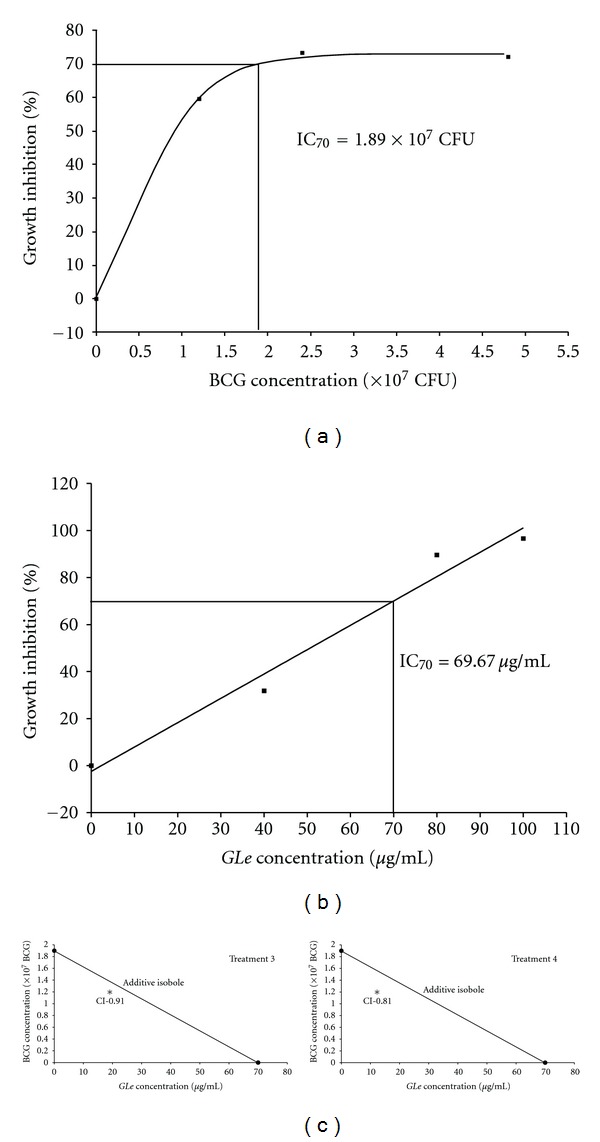
Dose-response curves of (a) BCG and (b) *GLe* generated by nonlinear regression, in order to define the sinGLe-agent effects as IC_70_. On day 6 following drug cessation, and since only selected BCG concentrations were tested, the BCG cytotoxicity showed ranged from 60–73% when compared with the solvent control (i.e., BCG 0 CFU on day 6). In order to minimize the error, IC_70_ was used for isobologram analysis. Isobolograms (c) were plotted for treatments 3 and 4 with the IC_70_ determined for BCG and *GLe*. Concentrations of *GLe* and BCG were reflected on *x*- and *y*-axes, respectively. Area below the additive isobole (the line joining IC_70,BCG_ and IC_70,*GLe*_) indicates synergistic interaction. Combination indices (CI) were calculated as CI = C_BCG,70_/IC_70,BCG_ + C_*GLe*,70_/IC_70,*GLe*_ according to Zhao et al. [14]. Specifically, C_BCG,70_ and C_*GLe*,70_ are the concentrations of BCG and *GLe* used in treatments 3 and 4 to achieve 70% drug effect. IC_70,BCG_ (fixed as 1.2 × 10^7^ CFU for all experiments) and IC_70,*GLe*_ (19.12 *μ*g/mL in treatment 3; 12.37 *μ*g/mL in treatment 4) are the concentrations for sin*GLe* agents to achieve the same effect.

The interaction between *GLe* and BCG on HUC-PC cells was evaluated by another two treatment schedules (treatment 3 and 4 illustrated in Figure 1). Irrespective of combination treatment with *GLe* (treatment 3) or pretreatment of *GLe* (treatment 4), the growth inhibitory activities induced by 1.2 × 10^7^ CFU of BCG were significantly enhanced. Cell growth was completely halted (maintaining the initial cell density) and was observed in the 24-hour combination treatment of 80 *μ*g/mL *GLe* with 1.2 × 10^7^ CFU BCG (Figure 3(a)). In the following 5 days when the treatment was removed, viable cell numbers were reduced below the initial cell density by all test concentrations, of which 6%, 65%, and 80% of the HUC-PC cells were eliminated by combining 1.2 × 10^7^ CFU BCG with 40, 80, and 100 *μ*g/mL of *GLe*, respectively. On the other hand, cytotoxic activities of BCG were remarkably promoted and fastened by the pretreatment with *GLe* (treatment 4; Figure 3(b)). About 25% and 40% of the initial cell densities were eliminated by the pretreatment with 80 and 100 *μ*g/mL of *GLe*, respectively, immediately after complete cessation of BCG. Such cytotoxic effects were further progressed in the next 5 days, with about 60% cell reduction by 40 or 80 *μ*g/mL *GLe* and 73% cell elimination by 100 *μ*g/mL *GLe*. To confirm the relationship between *GLe* and BCG, combination index (CI) values were determined for treatment groups 3 and 4, where CI < 1 indicates synergism, CI > 1 indicates antagonism, and CI = 1 indicates additive effect. Our results (Figure 4(c)) clearly showed that HUC-PC cells treated with BCG showed synergistic loss of cell viability when combined with *GLe* (CI = 0.91) or pretreated with *GLe* (CI = 0.81).

### 3.3. Regulatory Activities of *GLe* on BCG-Mediated Cytokines

The coincubation of BCG and *GLe* (treatment 3) has promoted the secretion of IL6 dose-dependently and has diminished the production of IL-8 (statistically nonsignificant) and MCP-1 (*P* < 0.001) (Table 1). Oppositely, all BCG-mediated cytokines (IL-6, IL-8, and MCP-1) were dramatically (*P* < 0.001) suppressed by the 24-hour *GLe* pretreatment (Table 1). In particularly, MCP-1 level of HUC-PC cells in treatment 4 was dropped to nondetectable level when pretreated with 100 *μ*g/mL of *GLe*. However, IL-2 was not detectable in all cultures treated under treatment 3 or 4 schedules. 

## 4. Discussion

Current *in vitro* study reports encouraging findings that ethanol extract of *Ganoderma lucidum* exhibited synergistic cytotoxic effects with BCG on the adverse uroepithelial cells at premalignant stage, in addition to its regulatory effects on proinflammatory cytokines including IL-6, IL-8, and MCP-1. Further to our previously reported growth inhibitory properties of *GLe*, this is the first time to demonstrate that cytotoxicity of *GLe* or BCG was progressed continuously even after the treatment withdrawal. However, the BCG cytotoxicity exhibited on the HUC-PC cells was shown to be delayed after the BCG cessation. 

Previous studies on BCG cytotoxicity were mainly focused on high-grade tumor cells [15], which may not be able to reflect the actual clinical implications in removing the residual cells from the bladder wall. Apoptosis was induced on bladder cancer cell lines of grade 3 and 4 stages by the wall preparation of mycobacterium species [16]. In low-grade bladder tumor cell lines, cell cycle arrests were observed after exposing to BCG, but no DNA degradation was shown, and therefore, apoptotic effects could not be confirmed [17]. Besides, the authors postulated that those residual adverse cells following surgical ablation are premalignant rather than malignant, since patients are apparently cured with complete tumor foci clearance. Herein, BCG has only demonstrated at the lowest tested concentration at 1.2 × 10^7^ CFU a small reduction (about 15%) in viable cell number. Such cytotoxic effects were not observed at higher concentrations tested, in which the mechanism is uncertain. Action of BCG is believed to be relied on the inflammatory response upon urothelial internalization [18]. Subsequently, the expression of TNF-related apoptosis-inducing ligand (TRAIL/Apo2L) and Fas/CD95 ligand (FasL) was stimulated on infiltrated immune cells and Fas expression on tumor cells, resulting in tumor necrosis factor-alpha (TNF-*α*-) induced apoptosis [4, 19]. It was also supported by the findings that voided urine samples from TCC patients receiving BCG therapy were cytotoxic to RT-4 bladder cancer cells [4]. High levels of functional soluble forms of TRAIL were detected in such specimens [20]. In the present study, neither TNF-*α* nor IFN-*γ* was stimulated by the BCG treatment at the measured time point. However, FlowCytomix results revealed that IL-6 and IL-8 secretions were induced in the culture media of BCG-treated HUC-PC cells. IL-6 has evidenced for the ability in inducing interferon-gamma (IFN-*γ*), which is believed as a late responsive T-helper type 1 cytokine to stimulate the TRAIL expression, thus responsible for successful BCG prophylaxis [21, 22]. Thus, current findings speculate the delay of BCG cytotoxicity was due to the process of BCG internalization and subsequent immune response, which requiring further elucidation. 

On the other hand, activities of *GLe* have shown to be a fast action that early apoptotic events with positive annexin-V uptake were initiated at 3 hours during the 24-hour incubation course [11]. Consistent with the reported G2M phase cell arrest in HUC-PC and other TCC cell lines [10], results herein have further demonstrated the progressing cytotoxicity and driven the complete adverse cell clearance several days after treatment retraction, especially at dose 80 *μ*g/mL or above. However, before the *GLe* cytotoxicity can be translated into clinical applications, the specificity of such cytotoxicity to carcinoma or adverse cells must be defined. In preliminary studies, *GLe* has shown to exert mild cytotoxicity to normal uroepithelial (HUC-1) cells, but in a lesser extent than in the HUC-PC cells (data not shown), which suggests the selectivity on premalignant instead of normal urothelium. This is also consistent with the selective cytotoxic effects of *G. lucidum* on cancer cell lines versus normal cells in origins instead of urinary bladder [23]. Besides the antitumor activities, the administration of *G. ludicum* has increased the lifespan in tumor-bearing mice of mammary cancer and did not display any toxicity [24]. In human *in vivo*, *G. lucidum* did not cause toxic effects to harm the liver and kidneys of patients with breast cancer receiving conventional endocrine therapy, whereas the well-being and cancer-related fatigue were also improved [25]. 

Similar to the BCG activities, *GLe* was also shown to stimulate IL-6 and IL-8 in the HUC-PC cells. Induction of IL-6 secretion by other *G. lucidum* fractions has been reported from various cell types including human T cells and mouse splenocytes [26, 27]. The *GLe*-mediated IL-8 secretion was also correlated with neutrophilic chemotaxis attraction that may facilitate the apoptotic cell clearance [12]. Given that nuclear factor-*κ*B (NF-*κ*B) and activating protein-1 (AP-1) are the main signaling pathways responsible for cytokines induction, the expression of IL-6 mRNA in the Hu35E6E7 HUC cells was found to be exclusively triggered by BCG through the Toll-Like Receptor (TLR) signaling [28]. Whilst p50/65 NF-*κ*B activity was enhanced by *GLe* concurrent with the cytokine elevation in the HUC-PC cells [12]. Therefore, the next question we asked was whether *GLe* would interact with BCG to favor TCC prophylaxis and how. 

Based on the hypothesis that BCG induces interferon- (IFN-) medicated cytotoxicity for the residual cell clearance, a 50–60% initial response rate was evidenced in resistant and relapsing patients after receiving the intravesical instillation of IFN-*α*2B and reduced dose of BCG [29]. Antitumor activities on bladder cancer cells were demonstrated by the combination treatment of Maitake mushroom with IFN-*α* [30]. In the present study, the cytotoxic effects exhibited by *GLe* and BCG were shown to be additive when cells were coincubated with both drugs or preincubated with *GLe* first. Despite the fact that both *GLe* and BCG are immunologically active, in the HUC-PC cultures, pretreatment with *GLe* was shown to be suppressive on all BCG-mediated cytokines. However, IL-6 induced by BCG was further stimulated when coincubated with *GLe*, while IL-8 secretion was inhibited. Such results implied that the inactivation of cytokine induction did not compromise the overall cytotoxicity, suggesting that the BCG and *GLe* synergism was cytokine-independent. On the contrary, the reduction of cytokine could be explained by the dramatic cytotoxic effects that essential metabolisms are commonly ceased during cell termination [31]. This could be explained by the early priming of HUC-PC cells by *GLe* to undergo progressive apoptosis before BCG was added [11]. Coherently, the antineoplastic properties of BCG on TCC cell lines [32] and mice *in vivo* [33] were enhanced by a vascular endothelial growth factor (VEGF)—Sunitinib through apoptotic pathways. Besides the therapeutic efficacy, bacterial infection and subsequent inflammatory response following intravesical BCG instillation are also associated with the high side effect tolls [7]. Despite such adverse effects are frequently mild cases, with the commonest cystitis, fever and chills are at least partially linked with the induction of endogenous inflammatory cytokines in the bladder [5]. The incubation of NF-*κ*B, which is a transcriptional factor for many cytokines, including the IL-6 induced by *GLe* on HUC-PC cells [12], was shown to bear antitumor effects in other bladder cancer cells [34]. Interstitial cystitis is a form of inflammatory hypersensitivity characterized with high levels IL-6 and histamine in patients' urine [35]. This imposes another question whether the regulatory roles of *GLe* on BCG-mediated IL-6 and IL-8 would reduce also the side effects of BCG? However, more in-depth investigations are required using animal models. 

Furthermore, MCP-1 expression of the HUC-PC cultures were dose-dependently inhibited by the treatments of BCG or *GLe* alone, combination therapy of BCG and *GLe*, and *GLe* pretreatment followed by BCG. It has reported that MCP-1 levels produced in the urine have directly correlated with the bladder cancer stages and grades [36]. Contradictorily, MCP-1 was elevated in the serum and bladder biopsy of bladder cancer patients following BCG immunotherapy [37]. The chemoattracting properties of MCP-1 were believed to be involved for tumor eradication; however, more recent studies have also demonstrated the roles of MCP-1 in angiogenesis and promoting tumor progression [11]. The pro- and antitumor effects of MCP-1 remain controversial, but facts presented in this study have indicated that the premalignant HUC-PC cells were capable for MCP-1 production, whereas in response to *GLe*, the levels were significantly suppressed concurrently with the growth inhibition. The degree of MCP-1 suppression was clearly correlated with the cytotoxicity exerted by the combination therapy and pretreatment schedule. 

## 5. Conclusions

Novel findings have demonstrated the possible synergistic interaction between BCG and *GLe*, in terms of adverse TCC cells elimination. Both BCG and *GLe* were immunological active in the HUC-PC cells. However, the BCG-provoked cytokines were inhibited when the cells were first preincubated with *GLe*, proposing the potential implications in reducing the inflammatory-related toxicity of BCG and warranted more in-depth elucidation. 

## Figures and Tables

**Table 1:** Yuen et al.

Treatment schedule		Cytokine level (Mean ± SD); *n* = 3			
		IL-2 (pg/mL)	IL-6 (pg/mL)	IL-8 (pg/mL)	MCP-1 (pg/mL)
Treatment 1 (24 hours)					

BCG alone	0 CFU^†^	N.D.	76 ± 21	744 ± 193	1812 ± 124
	1.2 × 10^7^ CFU	N.D.	380 ± 25***	1629 ± 416***	1850 ± 258
	2.4 × 10^7^ CFU	N.D.	492 ± 29***	1580 ± 503***	1661 ± 278
	4.8 × 10^7^ CFU	N.D.	498 ± 35***	1651 ± 708***	1490 ± 170***
			(*F* = 1093)	(*F* = 20.82)	(*F* = 5.044)

Treatment 2 (24 hours)					

*GLe* alone	0 *μ*g/mL^†^	6 ± 11	57 ± 24	645 ± 102	1765 ± 115
	40 *μ*g/mL	7 ± 13	331 ± 37***	1581 ± 367***	1254 ± 101***
	80 *μ*g/mL	10 ± 19	822 ± 35***	2430 ± 397***	740 ± 71***
	100 *μ*g/mL	22 ± 29	1343 ± 130***	3091 ± 672***	671 ± 62***
		(*F* = 1.238)	(*F* = 541.6)	(*F* = 105.2)	(*F* = 280.6)

Treatment 3 (24 hours)					

BCG + *GLe *	0 *μ*g/mL^†^	N.D.	413 ± 202	1835 ± 808	1789 ± 308
1.2 × 10^7^ CFU	40 *μ*g/mL	N.D.	752 ± 39*	1733 ± 469	520 ± 137***
	80 *μ*g/mL	N.D.	754 ± 83***	1504 ± 328	818 ± 169***
	100 *μ*g/mL	N.D.	891 ± 40***	1436 ± 338	542 ± 101***
			(*F* = 11.12)	(*F* = 1.628)	(*F* = 42.89)

Treatment 4 (48 hours)					

24-hr *GLe *→ 24-hr BCG	0 *μ*g/mL^†^	N.D.	640 ± 44	2256 ± 830	3471 ± 338
1.2× 10^7^ CFU	40 *μ*g/mL	N.D.	270 ± 27***	874 ± 68***	962 ± 107***
	80 *μ*g/mL	N.D.	164 ± 31***	524 ± 243***	89 ± 103***
	100 *μ*g/mL	N.D.	161 ± 20***	634 ± 206***	N.D.***
			(*F* = 827.2)	(*F* = 43.45)	(*F* = 269.3)

The secretion of cytokines detectable in the conditioned media collected from the cells treated with different treatment schedules (N.D.: non-detectable; *P< 0.05; ***P< 0.001). Statistical significances of parameters in each treatment schedule group were compared with corresponding control^†^. Controls for treatment 1 and 2 were not identical, because the 0 CFU BCG in treatment 1 contains 33% (v/v) BCG diluents, whereas the 0 *μ*g/mL *GLe* in treatment 2 contains 0.1% (v/v) ethanol. Additionally, the diluting effect of BCG diluents in the complete media was suspected to be the cause of non-detectable level of IL-2 in treatment 1, as compared with the trace level produced in the *GLe* control in treatment 2. However, the secretion of other cytokines (IL-6, IL-8 and MCP-1 were not in trace amounts) was seemed to be not being affected by such dilution. *F* value of each treatment group was determined by one-way ANOVA, and Dunnett's post test was followed to determine *P* values.
